# Efficacy, Feasibility, Adherence, and Cost Effectiveness of a mHealth Telerehabilitation Program in Low Risk Cardiac Patients: A Study Protocol

**DOI:** 10.3390/ijerph18084038

**Published:** 2021-04-12

**Authors:** José-Manuel Pastora-Bernal, Joaquín-Jesús Hernández-Fernández, María-José Estebanez-Pérez, Guadalupe Molina-Torres, Francisco-José García-López, Rocío Martín-Valero

**Affiliations:** 1 Department of Physiotherapy, Faculty of Health Science, University of Granada, 18071 Granada, Spain; 2 Department of Nursing and Physiotherapy, Faculty of Nursing and Physiotherapy, University of Cadiz, 11009 Cadiz, Spain; joaquinjesus.hernandez@uca.es; 3 Department of Physiotherapy, Faculty of Health Science, University of Malaga, 29071 Málaga, Spain; mariajoseestebanezperez@gmail.com; 4 Department of Nursing Science, Physiotherapy and Medicine, University of Almeria, 04120 Almeria, Spain; guada.lupe@ual.es; 5 Department of Physiotherapy, University of Osuna, 41640 Seville, Spain; fjgarlop@gmail.com

**Keywords:** telerehabilitation, physical therapy modalities, cardiovascular diseases, telemedicine, cost-benefit analysis

## Abstract

Individual and group cardiac rehabilitation (CR) programs reduce cardiovascular morbidity and mortality by reducing recurrent events, improving risk factors, aiding compliance with drug treatment, and improving quality of life through physical activity and education. Home-based programs are equally effective in improving exercise capacity, risk factors, mortality, and health-related quality of life outcomes compared to hospital-based intervention. Cardio-telerehabilitation (CTR) programs are a supplement or an alternative to hospital rehabilitation programs providing similar benefits to usual hospital and home care. Despite this statement, implementation in the public and private healthcare environment is still scarce and limited. The main objective of this research was to evaluate the efficacy, feasibility, and adherence of a personalized eight-week mHealth telerehabilitation program in low-risk cardiac patients in the hospital of Melilla (Spain). The secondary aims were to investigate patient satisfaction, identify barriers of implementation and adverse events, and assess cost-effectiveness from a health system perspective. A study protocol for a single center prospective controlled trial was conducted at the Regional Hospital of Melilla (Spain), with a sample size of (*n* = 30) patients with a diagnosis of low-risk CVD with class I heart failure according to NYHA (New York Heart Association). Outcomes of this study, will add new evidence that could support the use of CTR in cardiac patients clinical guidelines.

## 1. Introduction

Effective secondary prevention programs have great potential to reduce the burden of cardiovascular disease (CVD). The *Global Burden of Disease* (GBD) study, published in 2018, revealed the worrying dimension of the CVD pandemic [1]. CVD caused one third of all deaths in 2015, with an estimated prevalence of 422 million cases, predominantly atherosclerotic, cardiovascular disease (coronary heart disease and stroke) [1]. In recent decades, CVD mortality rates have fallen in developed regions. Improvements in cholesterol levels, blood pressure and smoking are responsible for 44% of the decline, while 47% of the decline is due to evidence-based medical care, therapies, and surgery [2]. The American Heart Association (AHA), proposed and developed a new metric of ideal cardiovascular health index as a means of assessing the cardiovascular health of a population, in what they called “LS7” (Life simple seven). The system is based on the ideal values of seven cardiovascular risk factors and health behaviors, all of which are modifiable. The LS7 score includes seven risk control factors, including three health factors (blood pressure BP, total cholesterol, and blood glucose) and four health behavioral factors (body mass index (BMI), smoking, physical activity, and diet) [3].

Individual and group cardiac rehabilitation (CR) programs, reduce cardiovascular morbidity and mortality by reducing recurrent events, improving risk factors, aiding compliance with drug treatment, and improving quality of life through physical activity and education [4]. According to the clinical practice guidelines for stable cardiovascular patients of the European Society of Cardiology (ESC), secondary prevention through a cardiac rehabilitation program is highly recommended [4]. A recent Cochrane systematic review with meta-analysis including 12 articles, concluded that home- and center-based forms of cardiac rehabilitation seem to be equally effective in improving clinical and health related quality of life outcomes in cardiac patients [5]. Unfortunately, despite these benefits and recommendations, many heart patients do not participate in group or individual cardiac rehabilitation (CR) programs. According to the EUROASPIRE questionnaire, only 44.8% of patients after a coronary event or revascularization stated that they had been advised to attend CR programs, and of these, only 81.4% had attended CR programs (36.5% of all patients) [6].

A recent systematic review collecting evidence published between 2001 and 2016 suggested that cardiac rehabilitation is cost-effective, especially with physical exercise as a major component [7].

Moreover, economic evaluations of health interventions, provide important information to health care providers, patients, planners, and politicians on the availability of alternatives in the context of limited resources [8]. The World Health Organization (WHO) recommends the use of the value-for-money threshold to identify the cost-effectiveness of an intervention [9]. The American College of Cardiology (ACC) and the American Heart Association (AHA) recommended a more recent guideline for the evaluation of interventions in cardiac patients. This “value for money” for health is generally identified as ‘very good’, ‘relatively good’, or ‘not good’ [10]. The absence of major differences in the costs of a home versus hospital-based CR programs, as found in the 2015 Cochrane review by Taylor et al., support the continued expansion of home cardiac rehabilitation programs [11].

The European Society of Preventive Cardiology recommends the inclusion of cardiac telerehabilitation (CTR) programs as novel intervention strategies. They identify CTR as a supplement or an alternative to hospital programs [12]. Telerehabilitation (TR) programs for patients with heart conditions provided similar benefits to usual hospital and home care, with no adverse effects reported according to a recent systematic review in 2016 [13]. Telerehabilitation is a term used to describe the provision of rehabilitation services at a distance, using communication technologies [14]. The Global Observatory for eHealth defined mHealth or mobile health as medical and public health practice supported by mobile devices, such as mobile phones, patient monitoring devices, personal digital assistants, and other wireless devices [15]. Mobile technology also has the potential to overcome barriers in performing cardiac rehabilitation monitoring, becoming a useful tool to promote compliance. Studies have shown that the use of mobile health interventions has positive benefits in increasing participation in rehabilitation [13]. 

Despite the fact that CTR has been shown to be as effective as CR in the hospital and home environment in terms of exercise capacity and quality of life, to be cost-effective and to improve patient satisfaction and adherence, its implementation in the public and private healthcare environment is still scarce and limited [4,16,17]. Considering the current health era and the large number of patients with cardiovascular diseases, it is advisable to carry out feasibility, safety, and effectiveness studies in the implementation of cardiac telerehabilitation programs both in the hospital environment and in other areas of care.

The aim of this research was to evaluate the efficacy, feasibility, and adherence of a telerehabilitation program in low-risk cardiac patients in the hospital of Melilla (Spain).

The secondary aims of this trial were to investigate patient satisfaction, to identify barriers of implementation and adverse events, and to assess cost-effectiveness from a health system perspective (costs of health interventions).

## 2. Material and Methods

This piece of research is a single center prospective controlled trial conducted at the Regional Hospital of Melilla (Spain), which will facilitate voluntary participant recruitment and the supervised CTR program component.

This research involves the departments of Cardiology, Rehabilitation, Physiotherapy, and Research at the University of Granada. All CTR staff will receive training on how to integrate the intervention training. As this study runs parallel to clinical practice, all medical management, including medication prescription, will be at the discretion of the treating physician. All changes will be documented and considered during analysis. 

**Figure 1:**
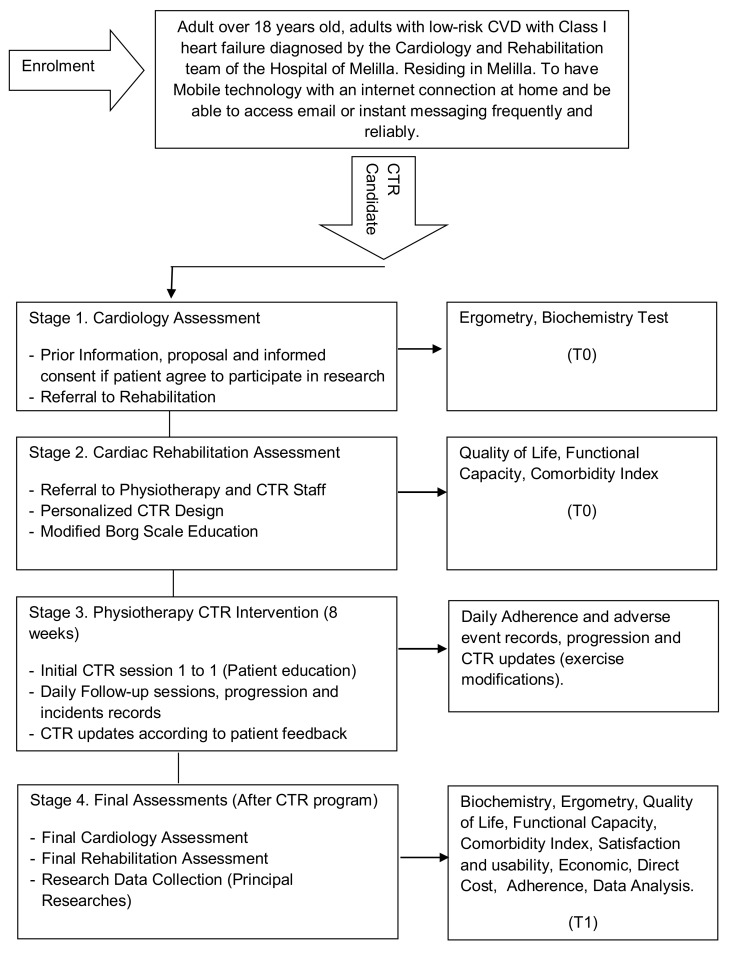
Study design. Abbreviations CVD, cardiovascular disease; CTR, cardio-telerehabilitation.

This research uses the guidelines on Standards for Quality Improvement and Excellence in Reporting (SQUIRE) [18] and will be carried out in accordance with CONSORT (Consolidated Standards of Reporting Trials) criteria [19]. A Standard Protocol Items Recommendations for Interventional Trials (SPIRIT) checklist is provided as Supplementary File S1, and the flow diagram for the study protocols is included as Figure 1.

### 2.1. Patients

The trial includes adults with a diagnosis of low-risk CVD with class I heart failure according to the NYHA (New York Heart Association) functional assessment criteria (24), diagnosed by the Cardiology and Rehabilitation team of the Hospital of Melilla. Patients must live in Melilla during the intervention phase, have mobile technology with an internet connection at home (including one of the following devices: desktop personal computer, laptop, tablet, or smartphone), and be able to access email or instant messaging frequently and reliably. Patients who suffered non-mild cardiac events prior to the first contact with this research, those whose pathologies are not classified as CVD, and those subjects who are not in full cognitive capacity that allows them to use new technology tools will be excluded. Patients will be excluded if they have any absolute or relative contraindications to exercise testing as per the American Heart Association guidelines [20]. Inclusion and exclusion criteria is show in Table 1.

In order to generate sufficient data for the development of this research, a sample size of (*n* = 30) patients will be recruited. The primary aims of this sample size determination is to evaluate whether the proposed intervention is feasible, safe, and effective. A larger randomized controlled trial that would have adequate power could be designed based on what was learned in this study. The strategies to achieve an adequate inclusion of participants that reach the target sample size include the multidisciplinary collaboration of the team from the Regional Hospital of Melilla (Cardiology Department, Head of the Rehabilitation Service and the Physiotherapy team). The collaborators were informed about the characteristics of the study in personal interviews and presentation. We ensured that the recruitment of patients has a socio-demographic diversity in relation to their social origin, gender, ethnicity, and education adapted to the particularities of the reference population. For the development of this research, an intentional non-probabilistic type of sampling will be used for the convenience of the study, due to the characteristics of the subjects. This trial has the approval of the Andalucía Ethics Committee with HIP version 17112020.

### 2.2. Intervention

Participants are assigned to the cardiac telerehabilitation group to receive an initial assessment. The CTR group receives a personalized 8-week exercise program through a mHealth application that allows health professionals to generate videos, images, and parameters of each exercise, as well as send them by email, instant messaging, and follow up on patients. In this study, we use the Physiotec exercise prescription app as it allows us to create personalized CTR programs according to particular clinical case and follow their progress. The CTR program describes the exercises to be performed, the number of repetitions according to the level of training and the criteria for progression. The CTR program includes aerobic exercise at 60–85% of the maximum heart rate (HR) reached in the ergometry test. As patients are taking β-blocker drugs, they will never reach their maximum HR. Patients will be instructed in the use of the Modified Borg Scale to assess their level of fatigue while performing the CTR program. Exercises customized to each participant should be performed between values 4–6 on this scale (out of a maximum of 10).

The type of exercise can be continuous or intervallic (load or HR controlled):

Continuous Exercise: After an initial warm-up period of 5–10 min, the patient is brought into a phase where he or she remains at a HR or resistance previously determined by the intervention team.

Interval Exercise: After an initial warm-up period, an exercise is started which alternates load peaks with lower intensity ones.

All patient training sessions should end with a return to the initial baseline situation (relaxation phase) of similar duration to the warm-up phase 5–10 min.

Typical CTR training session:

- Control of basal constants (by the patient): HR, blood pressure, weight, blood sugar…
- Permanent monitoring during the whole CTR session (patient must understand and follow the modified Borg scale as well as to use activity bracelets, pulse meters, or other monitoring instruments)
- Exercise session:
  ∘ Initial warm-up: 5–10 min (follow-up the video exercises provided through the CTR tool).
  ∘ Aerobic training: 35–45 min (follow up the video exercises provided through the CTR tool)
  ∘ Return to calm, relaxation-cooling: 5–10 min (follow up of the video exercises provided through the CTR tool)
  ∘ Recording of incidents and patient feedback in the CTR tool. Patient/physiotherapist feedback

Patients are initially supervised by the research team who will conduct 1-to-1 training sessions to ensure proper execution of the exercises and encourage patient adherence. Patients are instructed to perform self-training by following the video exercises through the CTR program. At the beginning of the study, advice is given on general care, in physical activity and issues concerning drug intake.

Patients are advised to refrain from any other specific training during the intervention period. Any deviations from the adherence and practice of the CTR program are recorded daily, noting any adverse incidents.

The TR group receives a personalized program for 8 weeks, including at least one session per day and is done through a web and mobile application. The telerehabilitation application allows health professionals to create personalized exercise programs, hold video conferences with patients, generate videos, images and parameters of each exercise, as well as send them by email and follow up patients through the mobile application.

### 2.3. Outcome Measures

Affiliation Data and Socio-Demographic Questionnaire: including age, gender, location, and other socio-demographic variables.

#### 2.3.1. Main Explanatory Variable

- Biochemical outcomes: obtained by blood analysis performed at the Hospital of Melilla including the following measures: red blood cell levels, glucose, creatine kinase (CK), triglycerides, cholesterol, high-density lipoprotein cholesterol (HDL-C), low-density cholesterol (LDL-C), and glycosylated hemoglobin (HbA1c).
- Cardiac function: obtained by performing an ergometry test at the Hospital of Melilla, including the following measurements: metabolic equivalent (MET), resting heart rate (HRrest), maximum heart rate (HRmax), and final heart rate (HRfinal), rating of perception of exertion scale (RPE), systolic blood pressure (SBP), diastolic blood pressure (DBP).
- Quality of life: obtained through the completion of self-reported questionnaires with the advice of the research team. The assessment of health-related quality of life will be assessed by the following two questionnaires: SF-12 and EuroQoL-5d. The SF-12 questionnaire is the short version of the SF-36, containing twelve questions from the SF-36. [21]. The SF-12 is an appropriate instrument of choice and is a practical alternative for measuring the general health status of the population [22]. The SF-12 questionnaire assesses eight dimensions of health-related quality of life: physical function, physical role, bodily pain, general health, vitality, social function, emotional role, and mental health [21]. Two sum scores are also obtained for the physical health component and the mental health component of the person. Thus, the sum index of the physical and mental component corresponds to the overall idea of general health. The EuroQoL-5D Questionnaire (EQ-5D) contains a descriptive system of one’s health status measured in five dimensions (mobility, self-care, activities of daily living, pain, and anxiety/depression) [23]. The EQ-5D has a visual analog scale (VAS) that assesses health status “on the present day” with scores ranging from 0 (worst imaginable health status) to 100 (best imaginable health status). The EQ-5D questionnaire provides information on the level of the problem (no problem, somewhat/moderate problem or severe problem) [24]. It has been shown to be a tool with an acceptable validity, with an estimated mean of 0.87 [25].
- Functional capacity: For the assessment of functional capacity we will use the Duke Activity Status Index (DASI). It is an assessment tool used to evaluate the functional capacity of patients with cardiovascular disease, such as coronary artery disease, myocardial infarction, and heart failure. It is a validated instrument and its Spanish version is available [26,27]. The original version include 12 questions on aspects of physical function related to activities of daily living. It allows a calculation of functional capacity reporting results in ml of O2/kg/minute with good correlation with peak VO2, and in turn can be compared with the METs [28].

#### 2.3.2. Secondary Explanatory Variable

Satisfaction and usefulness: The acceptance and usefulness of telemedicine applications is a prerequisite for identifying the potential clinical benefits of this technology. It is therefore important to complement this research with tools that examine patient satisfaction and perception [29]. An adaptation of the Telemedicine Satisfaction and Usefulness Questionnaire (TSUQ), whose psychometric analysis supports construct validity and internal consistency reliability, and which is available in English and Spanish, will be used [29]. The instrument used showed high reliability (Cronbach’s alpha 0.8) and evidence of validity with respect to perception in telemedicine [30]. It includes 17 questions that are assessed with a 5-point Likert subjective scale (1 totally disagree and 5 completely agree). The individual obtains scores from 17 to 85. The higher the score, the better the perception of the telerehabilitation intervention. Therefore each of the scores of the 17 variables indicated and finally the total score of the Test will be analyzed [29,30].

Analysis of the cost-effectiveness: To assess the cost-effectiveness of the telerehabilitation intervention, the international guidelines for conducting cost analyses in randomized clinical trials will be followed [31]. Such an economic analysis is based on the health sector perspective, which means that only costs corresponding to health interventions will be considered, and not related costs to the patient. Therefore, only costs related to the provision of health services will be taken into account [32].

Costs are divided into two categories of variables: firstly, costs related to clinical aspects (direct costs) and secondly, costs related to the use of the technology (indirect costs).

Direct costs: Numerical variable calculated on the basis of the number of hours of intervention and the cost of the physiotherapist according to the hourly wage in the health system in the centers where the research is carried out. It will be calculated based on the recording of the number of sessions in both groups.

Indirect costs: Numerical variable calculated based on the cost of using the technology for the telerehabilitation platform.

Adherence and safety: Participants will be advised to report and record barriers of use and any adverse event during the study. Number of sessions are automatically registered by the CTR tool.

The Charlson Comorbidity Index (CCI) will be included in the demographic, anamnesis and medical history data [33]. CCI is a system for evaluating life expectancy at ten years, depending on the age at which it is evaluated and the subject’s comorbidities. 

We will try to ensure that the sample data are similar at baseline and there are no significant differences in demographic, medical and other outcomes. The homogeneity of the sample will be analyzed and normality tests will be performed. An outline of the primary (4 dimensions) and secondary outcomes (3 dimensions) is shown at Table 2:

### 2.4. Data Collection Procedure, Monitoring, and Management

Once participants have been informed and agree to participate in the study, data will be collected for statistical analysis. This data collection will take place in the period February–June 2021. Initial assessment (Pre) and final 8-week assessment (Post) will be carried out by the research team with the cardiology, rehabilitation, and physiotherapy departments. The data will be aggregated to a research database created for this purpose and managed by the principal’s research using exportable data tables for statistical analysis. 

The research is designed in 4 stages, as presented in Figure 1 in a study design flow diagram:

Stage 1 includes 2 different processes: firstly the identification of candidates, the provision of prior information and the signing of informed consent in the event that the subjects agree to participate. Secondly, the assessments by the cardiology department with ergometry and biochemistry test at (T0-Pre). This stage ends with the referral to the rehabilitation department.

Stage 2 includes: the assessments by the rehabilitation department with quality of life, functional capacity outcomes, and comorbidity index added to previous clinical information. At this stage, the Modified Borg Scale Education is offered to participants to assess their level of fatigue during exercise. A personalized CTR program is proposed according to the Physiotherapy Department. This stage ends with the referral to the Physiotherapy department and CTR staff.

Stage 3 includes: 8 weeks CTR program with the supervision of the Physiotherapy Department and the CTR staff. An initial 1-to-1 session is offered with technology management and patient education. Daily follow up sessions include the progression, barriers of use, and adverse event records. CTR team will updates programs according to participant’s feedback and rehabilitation supervision.

Stage 4 includes: Final assessments and evaluation (T1-Post). At this stage, cardiology, rehabilitation, physiotherapy and principal research are compiled into the results of clinical and self-reported outcomes after 8 weeks CTR program including: biochemistry, ergometry, quality of life, and functional capacity. At this stage, satisfaction and usefulness, economic data safety, and adherence records will be added to research data’s for statistical analysis

### 2.5. Statistical Analysis

This research is a single center prospective controlled trial pre/post design. The results of the trial research will be presented as a summary of the outcome measures, together with the estimated effect size and precision. Statistical analysis will be performed according to the intention-to-treat principle. Patient characteristics will be summarized using frequencies and percentages for categorical factors, and using means and standard deviations for continuous measures in order to have as much information as possible available for exploration and analysis. The effect sizes will be calculated using Cohen’s d, so that the results can be compared to other studies. The outcome measures will be compared before and after the completion of the 8-week CTR program. All statistical analyses will be conducted using SPSS software. Statistical significance was set at *p* < 0.05.

## 3. Results

Enrollment began in February 2021. First study results will be reported in the middle of 2021. Recruitment is currently underway. The recruitment goal is 30 cardiac rehabilitation patients. Data collection is anticipated to be complete by July 2021.

If the results confirm beneficial effects in biochemical, cardiac function, quality of live, and functional capacity, the effectiveness of the intervention will be proven in patients with heart failure. The successful delivery of the intervention, the absence of significant adverse effects, and the user satisfaction will demonstrate the feasibility of these interventions in cardiac patients.

The results of this study will determine if a larger-scale intervention is feasible. Further, this pilot study will be the first to examine the effect of CTR intervention on patients in a cardiac rehabilitation setting in Melilla (Spain). The results of this study will provide differences compared to previous research: first of all, its implementation in the autonomous city of Melilla (located on the African continent with particular demographic characteristics); secondly the use of the innovative CTR platform based on the App Physiotec; thirdly, the collection of outcomes that combine clinical, self-reported and economic data and lastly, automated treatment adherence registry.

This study will add more evidence in support of the use of CTR program as an effective tool in new cardiac rehabilitation programs.

## 4. Discussion

Telemedicine has the promise of improving quality, increasing patient access, and reducing health care costs [34], and recent advances in telecommunications technologies have boosted the possibility of carrying out rehabilitation processes over the internet [35]. Studies have shown that telerehabilitation is effective in improving clinical outcomes in various pathologies, suggesting that increasing the intensity provided by telerehabilitation is a promising option to be offered to patients (32). This research should provide knowledge about the possibility of implementing CTR programs in the hospital environment, identifying the health resources and costs allocated to define new intervention policies in this group of patients. Unlike other studies that require software implementations in specific devices, our intervention generates few obstacles since it is available in any device that allows internet connection and that patients usually have (PC, laptop, tablet, smartphone), allowing access from any location and different devices prioritizing the use of mobile health. This contrasts with other studies that require a highly complex technological platform, software installation, and multidirectional cameras for controlled clinical control connecting the healthcare provider and the patient [36].

Technical problems (disconnection, device failures) and technological difficulties may arise in connection with the use of technology. However, staff members are available to provide technical support by phone or email, without the need to visit the patient at home to install or check any hardware. As possible adverse events, we considered the lack of improvement and positive evolution of the patient as well as the appearance of a low level of adherence. We also considered as an adverse event the performance of exercise with excessive workload. Patients will be informed of the importance of warning the health professional of any incident or setback in their recovery and their right to withdraw from participation in the research at any time.

Future lines of research would involve the development of clinical trials with a large sample size; a qualitative approach to attend focus groups to explore their thoughts and concerns about the intervention. Themes will be constructed from the data to give insight into the experiences of the different participant groups. As participation is important for rehabilitation departments, it is essential to include the participants’ perspective in the evaluation of the treatment. Finally, the opportunity to develop a multicenter randomized clinical trial with comparison groups performing in-hospital or home-based rehabilitation programs has the aim of identifying the most appropriate intervention.

## 5. Conclusions

This pilot study is the first to examine the effect of a CTR intervention on patients with heart failure in a cardiac rehabilitation setting in the Hospital of Melilla (Spain). Efficacy, feasibility, adherence, satisfaction, and cost-effectiveness must show if this study will add more evidence in support of the use of CTR program as an effective tool in new cardiac rehabilitation programs.

## Figures and Tables

**Table 1:** Inclusion and exclusion criteria.

Inclusions Criteria	Exclusions Criteria
Adult over 18 Years old	Cognitive ability not suitable for the use of technologic tools.
Diagnosis: Ischemic heart disease in New York Heart Association (NYHA) functional class I-II with preserved global systolic function or in intermediate range (LVEF > 40%) after revascularized acute coronary syndrome.	
Lives in Melilla during the research period.	Absolute or relative contraindications to exercise testing
To have mobile technology with an internet connection at home (including one of the following devices: desktop personal computer, laptop, tablet, or smartphone)	
Ability and knowledge to access email or instant messaging	

Abbreviations LVEF, Left ventricular ejection Fraction.

**Table 2:** Primary and secondary outcomes.

	Definition	Type
Primary Outcomes		
Biochemical	Hematies Level, Glucose, Creatine Kinase (CK), Triglycerides, Total Cholesterol, High Density Lipoprotein Cholesterol (HDL-C), Low Density Lipoprotein Cholesterol (LDL-C), Glycated Hemoglobin (HbA1c)	Clinical
Cardiac Function	Metabolic Equivalent (MET), Resting Heart Rate (RHR), Maximum Heart Rate (HRmax), Final Heart Rate (FHR), Rating Scale for Perceived Exertion (RPE), Systolic Blood Pressure (SBP), Diastolic Blood Pressure (DBP)	Clinical
Quality of Life	SF-12, EQ-5D	Self-Reported
Functional Capacity	Duke Activity Status Index (DASI)	Self-Reported
Secondary Outcomes		
Feasibility, Satisfaction and Usefulness	Telemedicine Satisfaction and usefulness Questionaire (TSUQ)	Self-Reported
Cost-Effectiveness	Direct Costs/Indirect Costs	€/Self-Reported
Adherence and Safety	Nº of sessions completed and adverse events record	Automatized App record

## Data Availability

The researcher declares that he follows the protocols of his work regarding the publication of data in accordance with the provisions of Organic Law 15/1999, of 13 December, on the Protection of Personal Data (LOPD), and that the data will be incorporated into a file for the purpose of carrying out this research project. Participating subjects will be informed of the possibility of exercising their rights of access, rectification, cancellation, and opposition of their data at the e-mail address provided by the principal investigator.

## Supplementary Materials

The following are available online at https://www.mdpi.com/article/10.3390/ijerph18084038/s1, File S1: SPIRIT 2013 Checklist: Recommended items to address in a clinical trial protocol and related documents.
